# Design and Implementation of a Power Semiconductor-Based Switching Mode Laser Diode Driver

**DOI:** 10.3390/mi15010031

**Published:** 2023-12-22

**Authors:** Chao-Tsung Ma, Fang-Yu Zhang

**Affiliations:** Applied Power Electronics Systems Research Group, Department of EE, CEECS, National United University, Miaoli City 36063, Taiwan; m1121002@o365.nuu.edu.tw

**Keywords:** high-power fiber laser, laser diode driver, power semiconductor device (PSD), full-bridge phase-shift converter

## Abstract

Fiber lasers are commonly used in many industrial applications, such as cutting, welding, marking, and additive manufacturing. In a fiber laser system, the driver of a pumping source using a laser diode (LD) module and its dynamic control capability directly affect the performance of the fiber laser system. The commercial design of pumping source drivers for high-power fiber lasers is mainly based on a linear-type DC power supply, which has two major drawbacks, i.e., lower efficiency and bulk. In this regard, this paper proposes for the first time a new design approach with a programmable switching mode laser diode driver using a power semiconductor device (PSD)-based full-bridge phase-shifted (FB-PS) DC-DC converter for driving a 200 W optical power laser diode module. In this paper, the characteristics of a laser diode module and the system configuration of the proposed laser diode driver are first introduced. Then, a current control scheme using the concept of phase angle shifting to achieve a fast dynamic current tracking feature is explained. The proposed current control technique with a fully digital control scheme is then addressed. Next, dynamic mathematical models of the laser diode driver system and controllers are derived, and the quantitative design detail of the controller is presented. To confirm the correctness of the proposed control scheme, a simulation study on a typical control case is performed in PSIM 9.1 software environment. To verify the effectiveness of the proposed LD driver, a digital signal processor is then used as the control core to construct a hardware prototype implementation for performing experimental tests. Results obtained from simulation and hardware tests show highly satisfactory driving performances in the laser diode’s output current command tracking control.

## 1. Introduction

The breakthrough in high-power fiber laser technology in recent years is an important milestone in the history of laser development. Fiber laser technology can be applied in many fields, including long-range wireless power transfer, communication, cutting, lithography, heat treatment, military weapons, and more. In particular, high-power lasers are currently used in thick steel welding for the manufacturing of vehicles, ships, and aircraft and the assembly of wind turbine discs and shafts. The advantages of laser welding include deep penetration, low heat input, high speed, fast cooling, and focused heating [1,2]. Laser welding is superior to electron beam welding because of its immunity against a magnetic field or vacuum environment, but it results in porosity in the object [3]. Laser paint stripping (LPS) allows non-contact paint and coating removal and is highly efficient, low-damage, simple to control, low-pollution, and immune to environmental issues [4]. Laser surface hardening (LSH) makes the surface of an object hard and wear-resistant while avoiding increased thickness. This technique results in low distortion of the hardened object, and it is fast, accurate, highly reproducible, and clean [5]. Three-dimensional printing, also known as additive manufacturing (AM), is highly competitive in the customization of products such as aerospace equipment, dental material, and microchannel heat sink. It is time- and cost-saving and low-waste [6,7,8,9]. Other important techniques include selective laser melting (SLM) [10] and laser metal deposition (LMD) [11].

Currently, most of the pumping sources adopt high-power semiconductor laser technology, also known as diode lasers, with wavelengths mostly in the range of 800 to 980 nm. High flexibility, durability, efficiency, reliability, and small size are the main advantages of a laser diode (LD). The quality and the control mechanism of an LD’s pumping source directly affect the performance of the LD’s optical output. Since an LD is easily damaged by overshoot or oscillation induced by switching devices, the design of the driving circuit is especially important. It is required that the driver supplies a smooth current. There are currently two driving modes for an LD: continuous mode (CM) and pulse mode (PM). In general, PM is more favorable because it yields greater efficiency. However, it is crucial to deal with overshoot and oscillation at the rising edge of a pulse, and the time delay between reference signal and actual output [12].

Complete discussions and design examples of the pumping sources required for fiber lasers are rarely found in the literature. At present, most of the patents on LD drivers (LDDs) are aimed at low-power applications; exclusive high-power LDD circuit design is even rarer. K. Jin and W. Zhou [13] reviewed recent progress of wireless laser power transmission. It was pointed out that conventional linear drivers which employ linear current regulators had low efficiencies and bulk volumes, and thus switched mode LDDs were a good alternative. It was also suggested that PM driving yielded a better performance. In [14], a Gallium Nitride (GaN) power transistor application used in driving high-performance lidar performed well, and it was mentioned that the two best LDDs for a lidar driving application are the capacitive discharge driver and the FET-controlled driver. The advantages of capacitive discharge drivers included no thermal runaway and a longer minimum pulse width, while the main advantage of FET-controlled drivers was the higher maximum pulse repetition frequency (PRF). The method of driving high-power LD arrays with a PM power supply was proposed in [14]. The power supply consisted of a capacitor and a linear current regulator, and the proposed simple “micro-current pre-start” control was effective for the elimination of overshoot and oscillation. A 25.6 kW prototype was built and used to verify the control method. W. Zhou and K. Jin [15] evaluated the efficiencies of CM driving and PM driving using a buck-boost converter and a buck converter in parallel for the LD module M1F4S22-808-50C-SS2.7 by DILAS, where PM yielded better performances in both simulation and implementation tests. In [16], a 50 A, 800 kHz PM current drive based on a single-stage switched-mode power supply (SMPS) was built for two 2 V LDs in series. The absence of linear stages reduced power loss. A GaN-based synchronous buck converter and a Si-based equivalent were studied in [17] for the GaN’s application in high-power LDDs. Losses were reduced by optimizing the dead-time, yielding highly promising results at a 700 kHz switching frequency in a 11 A, 300 W prototype.

This paper aims to present the design details of a 600 W continuous wave (CW) LDD. The Section 2 will first explore the characteristics and equivalent load of the tested LD module. The design of the LDD configuration and the required control algorithm will be explained in the Section 3. In the Section 4, the operating modes of the proposed driving circuit will be analyzed, and the control scheme will be explained. Then, the necessary mathematical models will be derived for further quantification design of the controller. Lastly, PSIM models of the complete LDD system along with its controller will be established and used for simulation studies in which a scenario with a series of step output variations is planned. Results obtained from experimental tests with the developed hardware will be presented in the Section 5. Finally, this paper will be concluded in the Section 6.

## 2. Characteristics and Equivalent Load of the LD Module

**Figure 1:**
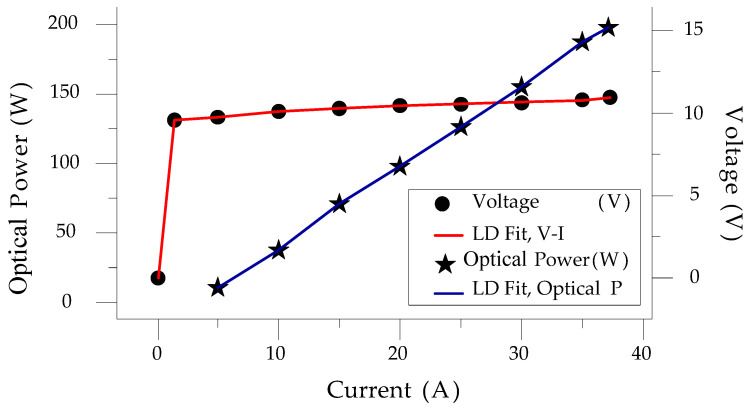
The V-I and optical power curves of a DILAS 200 W LD.

In the testing phase of LDD development, if an actual LD module is used, it is likely that the module will be damaged, and the price of a high-power LD module is very high. Therefore, this paper uses an equivalent load made up of a number of diodes connected in series and parallel in place of the LD. In addition, this method allows adjustable module parameters for adapting different LD specifications. Commercial LD modules come in a variety of types and with different V-I and P-I characteristics. In other words, there is no specific load model that can emulate all types of LD modules. At present, most of the related papers have focused on the LD’s performance and on how to improve the optical power or reduce the threshold current for LDs; the V-I characteristics and its equivalent circuit have not been discussed specifically. In this paper, we first use LD data from DILAS to carry out a characteristic curve analysis. An equivalent load is then built to replace the LD for follow-up implementation of this work. The characteristics of a general LD can be roughly described as shown in Equation (1). If we consider the equivalent resistance of the LD resonant cavity *r_s_*, Equation (1) can be expressed as Equation (2). Figure 1 shows the characteristics of a DILAS 200 W LD. It is noted that the power conversion efficiency of the DILAS LD module is about 47.5%. This means that an output of 100 W LD optical power requires about 200 W input of electrical power, as shown in Figure 1.

(1) $$I=I_{0}(T)\left[\exp(eV/nK_{B}T)-1\right];$$

(2) $$V=\left(nK_{B}T/e\right)\ln\left\{\left[I/I_{0}(T)\right]+1\right\}-Ir_{s},$$

where *I* represents LD current, *I*_0_(*T*) represents reverse saturation current, *n* represents the material ideal coefficient, *K_B_* represents the Boltzmann constant, *e* represents electron charge, *V* represents voltage across the LD, *T* represents ambient temperature, and *r_s_* represents resonant cavity equivalent resistance.

## 3. LDD System Configuration

**Figure 2:**
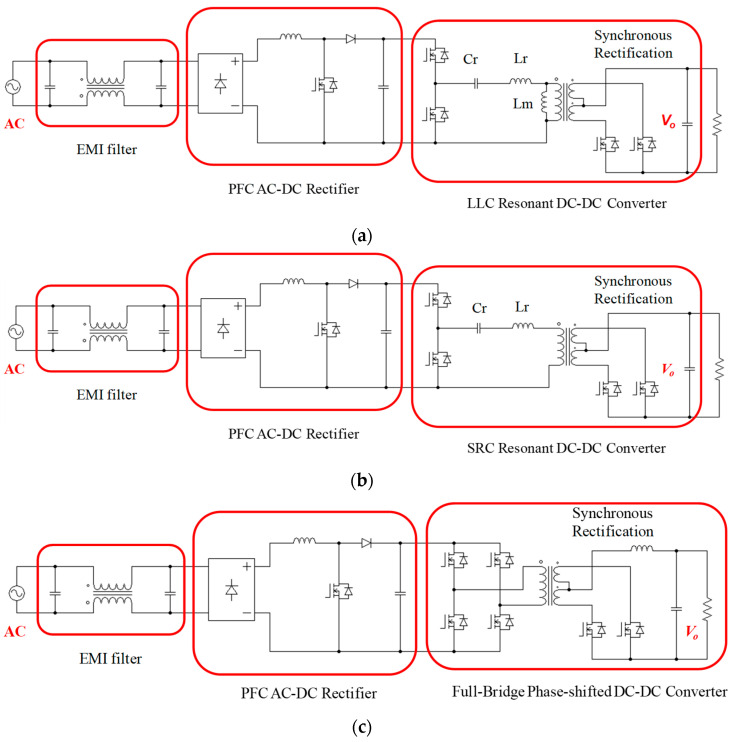
Schematics of feasible circuit configurations in LDD applications: (**a**) LLC DC-DC converter, (**b**) SR DC-DC converter, and (**c**) FB-PS DC-DC converter.

The V-I characteristics of an LD are similar to those of a general light-emitting diode (LED), so their driving and control techniques should also be similar, including a voltage clamp for protection and constant current control. However, the power of an industrial LD is much higher than that of a general LED, and the voltage output is low, so circuit configurations for general <200 W LEDs are not applicable in this case. The LDD driver presented in this paper is rated at 600 W, with the output voltage and current 12 V and 50 A, respectively, and is also commonly used in server power (SP) applications. Feasible configurations of a high-efficiency driving circuit topology in LDD applications include an LLC DC-DC converter, a series resonant (SR) DC-DC converter, and a full-bridge phase-shift (FB-PS) DC-DC converter, as shown in Figure 2. Because the output voltage requirement of the LD module is normally very low, in order to achieve high efficiency, the secondary side should adopt synchronous rectification. Furthermore, it is required that the output current is adjustable, and so resonant converters are less suitable because frequency adjustment is required to enable an adjustable current, which narrows the adjustment range of the system gain. Moreover, the efficiency of a resonant converter system will deteriorate when it deviates from its resonance point.

In this paper, the objective LD module demands a high current rise/fall rate (less than 1 ms). As a result, a fast current control technique is necessary. In this aspect, an FB-PS converter can adjust the current output to control the load power by adjusting the phase shift and duty ratio. It should be noted that the power flow control of the FB-PS DC-DC converter is within a single quadrant, which is less favorable for fast current-fall control of general loads; however, the LD module already has fast current-fall characteristics. Based on this, the FB-PS DC-DC converter topology was chosen as the proposed LDD circuit configuration. By controlling the phase shift angle between the leading leg and lagging leg, the LDD’s output power can be controlled as desired. Secondly, to improve the quality of current adjustment for the LD module, it is necessary for the control to incorporate parameter adaptation, which is, in fact, hard to achieve with an analog circuit. As a result, the first and second stage of the proposed LDD circuit are integrated and controlled with a fully digital control scheme. In addition to parameter adaptation, some energy management strategies can also be integrated to reduce power losses, meeting Energy Star’s energy consumption requirements.

## 4. FB-PS DC-DC Converter Design and Simulation

**Figure 3:**
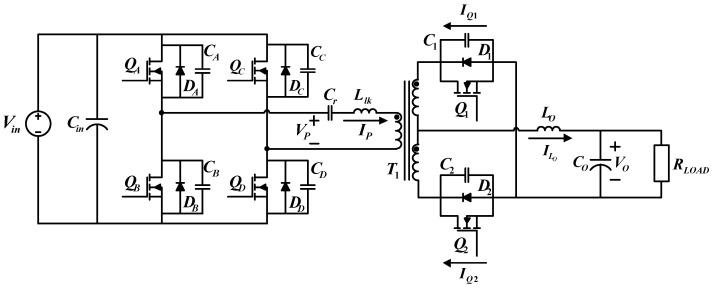
Schematic of the proposed LDD using the FB-PS DC-DC converter.

Figure 3 shows the circuit configuration of the proposed FB-PS DC-DC converter. The primary side is connected to a DC bus, normally the DC terminal of an AC/DC module, and the secondary side is connected to the LD module or its emulator. The main components of this circuit include four power switching devices, i.e., *Q_A_* to *Q_D_*, a center-tapped transformer, *T*_1_, two synchronous rectification switches, *Q*_1_ and *Q*_2_, a filter inductor, *L_o_*, and a capacitor, *C_o_*, and an external resonant inductor ( $L_{lk}$ ) and a capacitor ( $C_{r}$ ), which, with the parasitic capacitor of the power switch, form a resonant circuit, enabling zero-voltage switching (ZVS) of the power switches. The control method of FB-PS converters is different from that of conventional FB converters. The difference is that conventional FB converters adopt pulse width modulation (PWM), while for FB-PS converters the pulse phase modulation (PPM) is used in this design case.

To design a secondary side rectifier, this study adopts a center-tapped rectifier and replaces conventional rectifier diodes with low impedance MOSFETs to decrease power losses. The control of synchronous rectifier switches can be divided into two types: the first is self-driven control, where an auxiliary coil is added in order to directly drive the switches on the secondary side; the second method is through PWM. Considering the flexibility for future implementation, PWM is adopted in this paper.

### 4.1. Operating Status Analysis of the FB-PS DC-DC Converter

**Figure 4:**
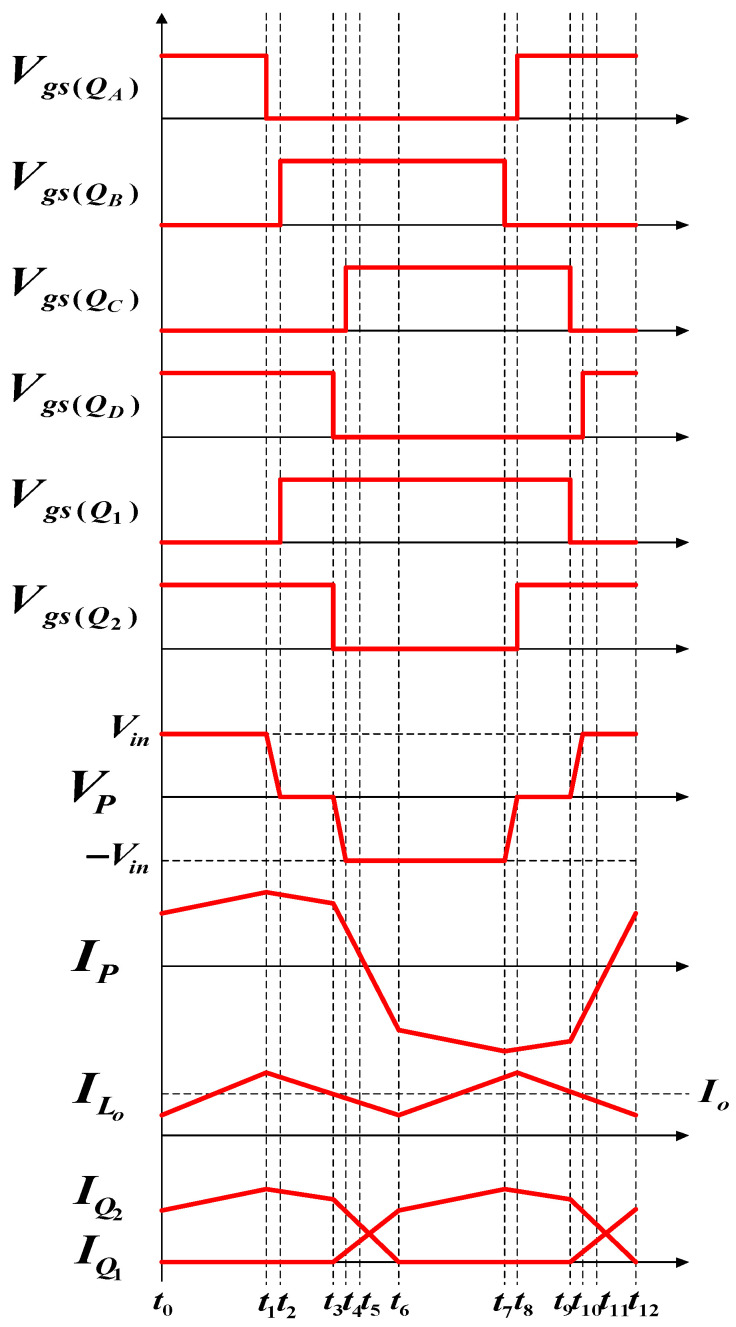
FB-PS converter operating waveforms including switch driving signals and transformer primary and secondary side voltages and currents.

Figure 4 shows the operating waveforms of the different signals of the FB-PS converter. In a complete switching cycle, there are twelve operating statuses according to the behaviors of the switching devices. However, the twelve operating statuses can be divided into two groups (statuses 1–6 and statuses 6–12) because they show a symmetrical form. Therefore, only statuses 1–6 are described here.

#### 4.1.1. Operating Status 1 (*t*_0_ − *t*_1_)

**Figure 5:**
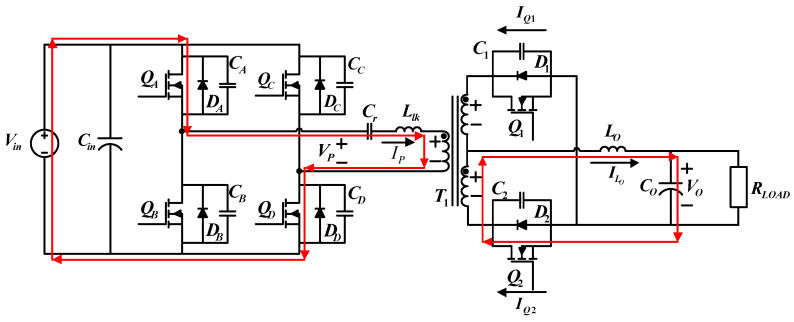
The paths of current flows during operating status 1.

During the first operating status, $Q_{A}$ and $Q_{D}$ on the primary side and $Q_{2}$ on the secondary side are on. Figure 5 shows the paths of current flows. Before *t*_0_, the transformer primary side voltage equals the input voltage (*V_P_ = V_in_*). During this interval, output inductor current *I_Lo_* gradually increases.

#### 4.1.2. Operating Status 2 (*t*_1_ − *t*_2_)

**Figure 6:**
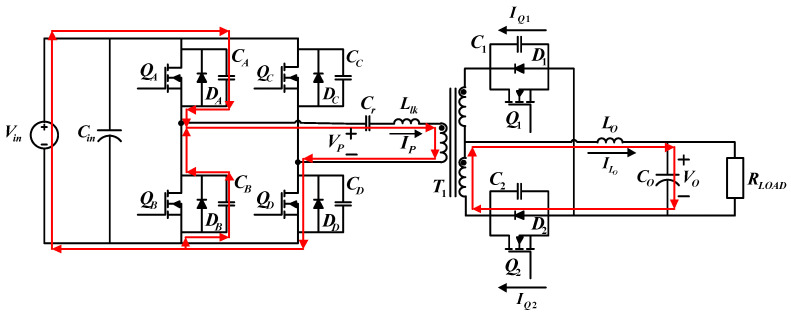
Current flows during operating status 2.

During the second operating status, only $Q_{D}$ on the primary side is on. Figure 6 shows the paths of current flows. At *t*_1_, energy stops flowing to the secondary side. However, the transformer and output inductor maintain the current flows. As a result, $L_{O}$ discharges, and thus the current gradually decreases.

#### 4.1.3. Operating Status 3 (*t*_2_ − *t*_3_)

**Figure 7:**
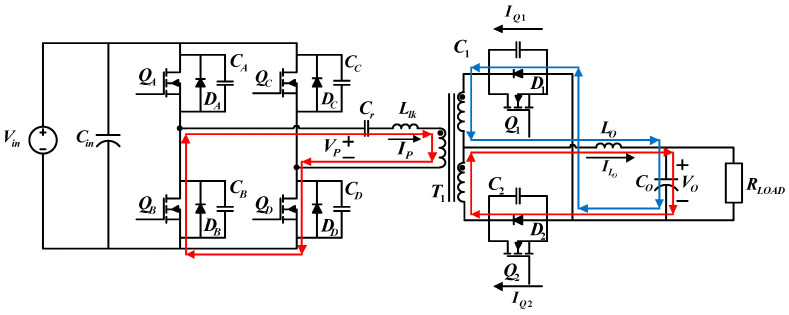
Current flows during operating status 3.

During the third operating status, $Q_{B}$ and $Q_{D}$ on the primary side are on. Figure 7 shows the paths of current flows. At *t*_2_, $Q_{B}$ will be turned on first. When *V_DS_* of $Q_{B}$ decreases to zero, $Q_{B}$ is turned on, achieving ZVS. During this period, *V_P_ = 0*, and the secondary side keeps freewheeling through $D_{1}$ and $D_{2}$ , while most of the current flows through $D_{2}$ and $I_{L_{o}}$ continues to decrease.

#### 4.1.4. Operating Status 4 (*t*_3_ − *t*_4_)

**Figure 8:**
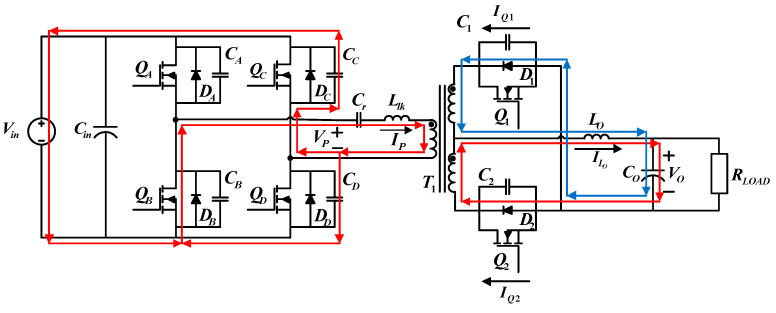
Current flows during operating status 4.

During the fourth operating status, only $Q_{B}$ on the primary side is on. Figure 8 shows the paths of current flows. At *t_3_*, $Q_{D}$ is off, so the current flows in $C_{D}$ and $C_{C}$ . During this period, *V_P_* equals the voltage across $C_{D}$ , so it increases from zero to *V_in_*, while the voltage across $C_{C}$ gradually discharges to zero. On the secondary side, the output inductor maintains the current flows, so $D_{1}$ and $D_{2}$ are both off. As in the previous period, *I_Lo_* gradually decreases, while *I_Q_*_2_ decreases and *I_Q_*_1_ increases.

#### 4.1.5. Operating Status 5 (*t*_4_ − *t*_5_)

**Figure 9:**
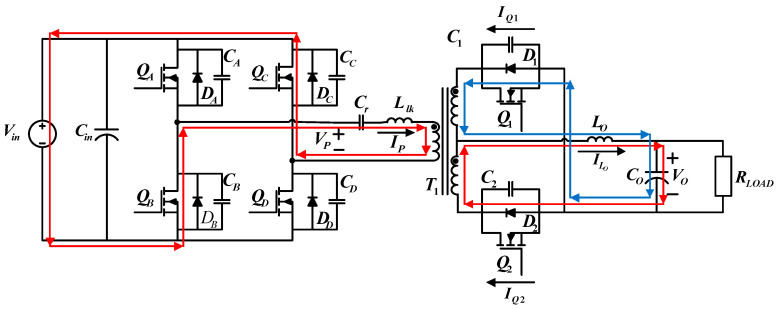
Current flows during operating status 5.

During the fifth operating status, $Q_{B}$ and $Q_{C}$ on the primary side and $Q_{1}$ on the secondary side are on. Figure 9 shows the paths of current flows. At *t*_4_, because the voltage across $C_{C}$ equals zero, $D_{C}$ is first turned on. When *V_DS_* has decreased to zero, a driving signal is inputted to turn $Q_{C}$ on, achieving ZVS. During this period, $V_{p}$ equals $-V_{in}$ . On the secondary side, the output inductor maintains the power flows, so $D_{1}$ and $D_{2}$ are both on, while $Q_{1}$ is on, and $Q_{2}$ is off. Because primary side current is not able to support the load current yet, $I_{L_{o}}$ gradually decreases, while $I_{Q_{2}}$ decreases, and $I_{Q_{1}}$ increases until the primary side current decreases to zero at *t*_5_.

#### 4.1.6. Operating Status 6 (*t*_5_ − *t*_6_)

**Figure 10:**
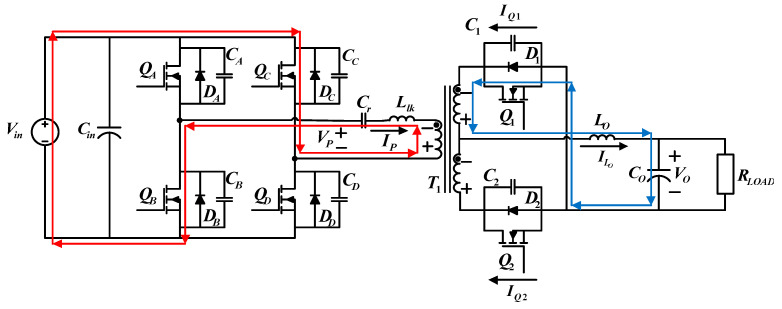
Current flows during operating status 6.

During the sixth operating status, $Q_{B}$ and $Q_{C}$ on the primary side and $Q_{1}$ on the secondary side are on. Figure 10 shows the paths of current flows. At *t*_5_, $I_{p}$ has decreased to zero and starts to become negative, and $V_{p}$ equals $-V_{in}$ . During this time, $Q_{B}$ and $Q_{C}$ are on, while $Q_{1}$ is on, and $Q_{2}$ is off. On the secondary side, the inductor continues to discharge, and *I_Lo_* continues to decrease and has completely switched from flowing through $Q_{2}$ to flowing through $Q_{1}$ .

### 4.2. Control Architecture

**Figure 11:**
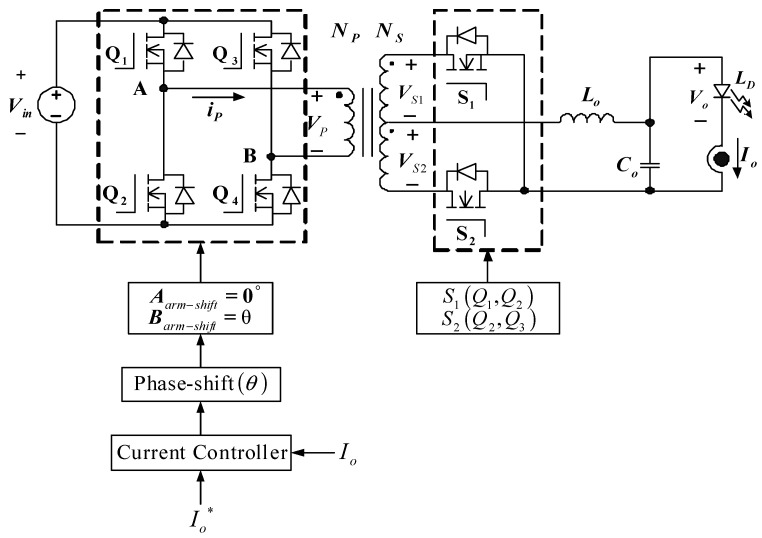
Signals and current control architecture of the proposed FB-PS converter.

The control architecture of the proposed FB-PS DC-DC converter is shown in Figure 11, where the turn ratio is defined as follows: *N* = *N_p_*/*N_s_*. The control of the two switching legs adopts phase shift control. The leading leg (leg A) is used as the preference phase (0°), and the trigger of the lagging leg (leg B) and its phase shift is produced by the proposed current controller. In this case, the load is an LD module; therefore only the phase shift is always larger than zero.

### 4.3. Transfer Function Derivation

**Figure 12:**
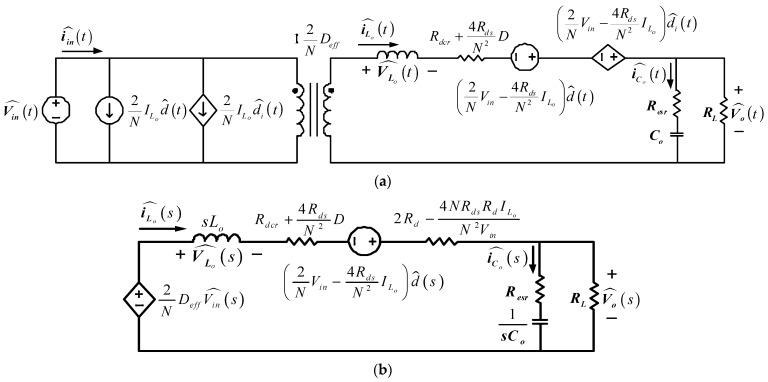
(**a**) AC signal model of FB-PS converter and (**b**) Laplace transform of the model.

The transfer function of the proposed FB-PS DC-DC converter can be obtained using small signal models derived from the main operating statuses, i.e., operating statuses 1–4 which cover all the converter’s dynamics in the switching operations of all power electronic switches. The complete system transfer function is also required to take into consideration how the resonant inductor and rectifier inductor affect the secondary side duty cycle. It should be noted that when the energy of the FB-PS DC-DC converter is transferred from the primary side to the secondary side of the high-frequency transformer, there will be a reduced working duty cycle (ΔD) due to the influence of the resonant inductance. This will affect the effective duty cycle (*D_eff_*) of the secondary side of the transformer. Taking the reduced duty cycle into account, the small signal model of the FB-PS DC-DC converter can be obtained. Refer to Figure 11 for the parameters and notations in the following derivation. Figure 12a shows the derived AC signal model of the FB-PS DC-DC converter, and Figure 12b shows the Laplace transform of the converter model. It is noted that the Laplace transform of the model shown in Figure 12b was taken at the secondary side of the transformer, with the primary side voltage reflexed to the secondary side. During operating status 1, VSD1 = VSD2 = VD. Primary and secondary side voltages, filter inductor voltage, capacitor current, and input current can be expressed as Equations (3)–(7), respectively.

(3) $$V_{p}=V_{in}-2R_{ds}\cdot i_{L_{o}}/N;$$

(4) $$V_{S2}=V_{p}/N=V_{in}/N-2R_{ds}\cdot i_{L_{o}}/N^{2};$$

(5) $$\begin{array}{l} V_{L_{o}}\left(t\right)=L_{o}\left[di_{L_{o}}\left(t\right)/dt\right]=-V_{D}+V_{S2}-V_{o}\left(t\right)\\ -R_{dcr}\cdot i_{L_{o}}\left(t\right)\\ =-V_{D}+V_{in}\left(t\right)/N-V_{o}\left(t\right)-R_{dcr}\cdot i_{L_{o}}\left(t\right); \end{array}$$

(6) $$i_{C_{o}}\left(t\right)=C_{o}\left[dV_{C_{o}}\left(t\right)/dt\right]=i_{L_{o}}\left(t\right)-\left[V_{o}\left(t\right)/R_{L}\right];$$

(7) $$i_{in}\left(t\right)=i_{L_{o}}\left(t\right)/N,$$

where *V_P_* represents primary side voltage, *V_in_* represents input voltage, *R_ds_* represents switch on resistance, *i_Lo_* represents output inductor current, *N* represents turn ratio, *V_S2_* represents secondary side voltage, *V_Lo_* represents output inductor voltage, *L_O_* represents output inductance, *V_D_* represents voltage across the body diode of S_2_, *V_o_* represents output voltage, *R_dcr_* represents DC resistance of the output inductor, *i_Co_* represents output capacitor current, *C_o_* represents output capacitance, *V_Co_* represents output capacitor voltage, *R_L_* represents load resistance, and *i_in_* represents input current. During operating status 2, filter inductor voltage, capacitor current, and input current can be expressed as Equations (8)–(10), respectively.

(8) $$V_{L_{o}}\left(t\right)=L_{o}\left[di_{L_{o}}\left(t\right)/dt\right]=-V_{D}-V_{o}\left(t\right)-R_{dcr}\cdot i_{L_{o}}\left(t\right);$$

(9) $$i_{C_{o}}\left(t\right)=C_{o}\left[dV_{C_{o}}\left(t\right)/dt\right]=i_{L_{o}}\left(t\right)-V_{o}\left(t\right)/R_{L};$$

(10) $$i_{in}\left(t\right)=0.$$

During operating status 3, filter inductor voltage, capacitor current, and input current equations are the same as during operating status 1. During operating status 4, according to the inductor volt-second balance law and the capacitor ampere-second balance law, all the above obtained equations are used to obtain the following average values. Here, we define the amount of time taken by statuses 1–4 in a switching cycle as *d*_1_(*t*) through *d*_4_(*t*), respectively. We know that *d*_1_(*t*) *+ d*_2_(*t*) *+ d*_3_(*t*)*+ d*_4_(*t*) *=* 1, and *d*_2_(*t*) *+ d*_4_(*t*) *=* 1 *− d*_1_(*t*) *− d*_3_(*t*). Then, we let *d*_1_(*t*) *= d*_3_(*t*) *= d*(*t*). As a result, Equations (8)–(10) are expressed as (11)–(13), respectively, and the average current in a switching cycle is expressed as Equation (14). The definition for each of the perturbation terms of the parameters can be found in Appendix A.

(11) $$\begin{array}{l} {\langle V_{L_{o}}\left(t\right)\rangle }_{T_{s}}=L_{o}d{\langle i_{L_{o}}\left(t\right)\rangle }_{T_{s}}/dt\\ =\left[-V_{D}+V_{in}\left(t\right)/N-V_{o}\left(t\right)-\left(R_{dcr}+2R_{ds}/N^{2}\right)i_{L_{o}}\left(t\right)\right]2d\left(t\right)\\ +\left[-V_{D}-V_{o}\left(t\right)-R_{dcr}\cdot i_{L_{o}}\left(t\right)\right]\cdot \left[1-2d\left(t\right)\right]\\ =2V_{in}\left(t\right)\cdot d\left(t\right)/N-4R_{ds}\cdot i_{L_{o}}\left(t\right)\cdot d\left(t\right)/N^{2}\\ -V_{D}-V_{o}\left(t\right)-R_{dcr}\cdot i_{L_{o}}\left(t\right)\\ =0; \end{array}$$

(12) $$\begin{array}{l} {\langle i_{C_{o}}\left(t\right)\rangle }_{T_{s}}=C_{o}d{\langle V_{C_{o}}\left(t\right)\rangle }_{T_{s}}/dt\\ =\left[i_{L_{o}}\left(t\right)-V_{o}\left(t\right)/R_{L}\right]d_{1}\left(t\right)+\left[i_{L_{o}}\left(t\right)-V_{o}\left(t\right)/R_{L}\right]d_{2}\left(t\right)\\ +\left[i_{L_{o}}\left(t\right)-V_{o}\left(t\right)/R_{L}\right]d_{3}\left(t\right)+\left[i_{L_{o}}\left(t\right)-V_{o}\left(t\right)/R_{L}\right]d_{4}\left(t\right)\\ =i_{L_{o}}\left(t\right)-V_{o}\left(t\right)/R_{L}=0; \end{array}$$

(13) $$\begin{array}{l} {\langle i_{in}\left(t\right)\rangle }_{T_{s}}=i_{L_{o}}\left(t\right)\left[d\left(t\right)+d\left(t\right)\right]/N\\ =2i_{L_{o}}\left(t\right)\cdot d\left(t\right)/N; \end{array}$$

(14) $$\begin{array}{c} {\langle i_{in}\left(t\right)\rangle }_{T_{s}}=i_{L_{o}}\left(t\right)\left[d\left(t\right)+d\left(t\right)\right]/N\\ =2i_{L_{o}}\left(t\right)d\left(t\right)/N \end{array}.$$

Next, to obtain the small signal model, the parameter’s perturbation terms are considered, higher order terms are ignored, DC terms are removed, and the duty cycle reduction, ΔD, is taken into consideration. This process yields the small signal model and the Laplace transform of the model in Figure 12. According to Figure 12b, Equation (15) can be obtained:

(15) $$\begin{array}{l} {\hat{V}}_{o}(s)/\hat{d}(s)\\ =R_{L}\left(2V_{in}/N-4R_{ds}\cdot I_{L_{o}}/N^{2}\right)\cdot \left(sC_{o}R_{esr}+1\right)/A, \end{array}$$

where

(16) $$\begin{array}{l} A=s^{2}C_{o}L_{o}\left(R_{L}+R_{esr}\right)\\ +s\left[C_{o}R_{L}\left(R_{dv}+R_{dsc}\right)+C_{o}R_{esr}\left(R_{dv}+R_{dsc}+R_{L}\right)+L_{o}\right]\\ +\left(R_{dc}+R_{dsc}+R_{L}\right), \end{array}$$

(17) $$R_{dv}=2R_{d}-4R_{ds}R_{d}I_{L_{o}}/NV_{in},$$

(18) $$R_{dsc}=R_{dcr}+4R_{ds}D/N^{2}.$$

*R_esr_* represents equivalent series resistance. Finally, the relationship between the duty cycle and output current can be described as follows:

(19) $${\hat{I}}_{o}\left(s\right)/\hat{d}\left(s\right)=\left(2V_{in}/N-4R_{ds}\cdot I_{L_{o}}/N^{2}\right)\left(sC_{o}R_{esr}+1\right)/A,$$

and the phase shift angle, *θ*, can be expressed as follows:

(20) $$\theta =\left[180/\left(T_{s}/2\right)\right]\cdot DT_{s}/2=180\cdot D.$$

### 4.4. Controller Design for the Proposed LDD

**Figure 13:**
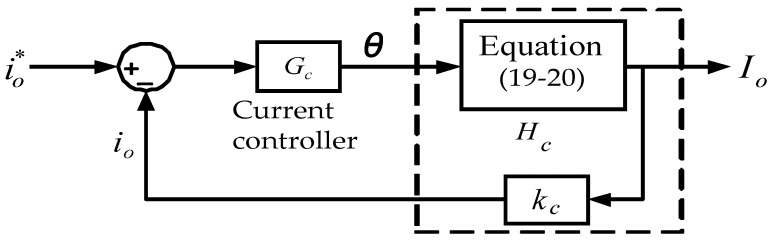
The proposed current controller.

In this design case, the proportional plus integral (PI) controller is used to design the current loop controller, *G_c_*(s). Here, the phase margin is designed at 76° and the crossover frequency *f_c_* is selected at 2.7 kHz. The designed current controller is shown in Figure 13. Here, *k_c_* is the sensing factor of the real-time output current (Io) of the FB-PS DC-DC converter. Some key design equations of controllers can be found in Appendix A. The objective of the designed current controller, *G_c_*, is to output a control signal that is equivalent to the phase shift (*θ*) of the controlled two switching legs of the FB-PS converter, according to the tracking error of the output current (Io), i.e., $\theta ={(i}_{o}^{*}-i_{o}).G_{c}$ .

### 4.5. Controller Quantification Design

**Figure 14:**
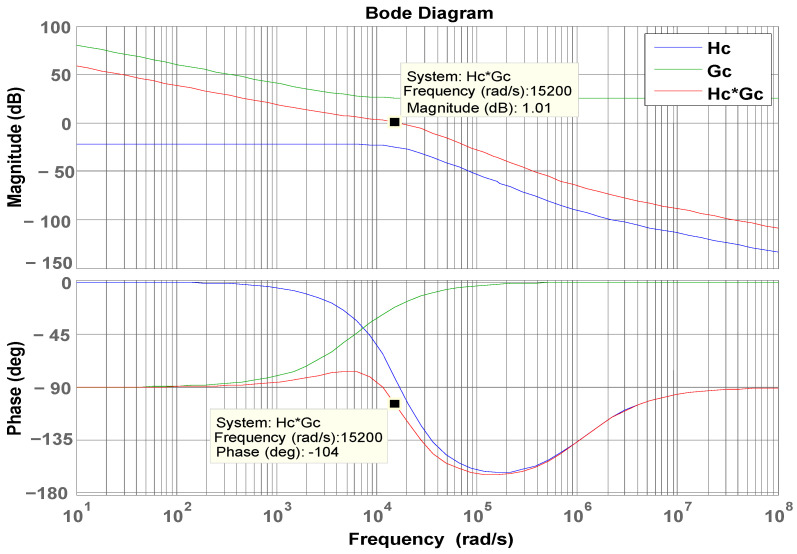
The current loop Bode plot.

The design specification of the proposed LDD with an FB-PS converter is as follows: DC 12 V output voltage, DC 390 V input voltage, 50 A rated output current, 600 W rated power, 65 kHz switching frequency, and 96% conversion efficiency. According to the previous derivation, the controller is designed as shown in Equation (21). The current loop Bode plot shown in Figure 14 verifies that the controller parameter satisfies the requirement for system stability and the dynamic specifications of the FB-PS converter. It is noted that a control system with good performance should have a phase margin above 45°. In this study, the PM of the current controller is finally designed at 76° to achieve the stability and dynamic specification set for the proposed FB-PS DC-DC converter.

(21) $$G_{C}=17.9483\left(s+5.987k\right)/s.$$

### 4.6. PSIM Simulation of the FB-PS DC-DC Converter

**Figure 15:**
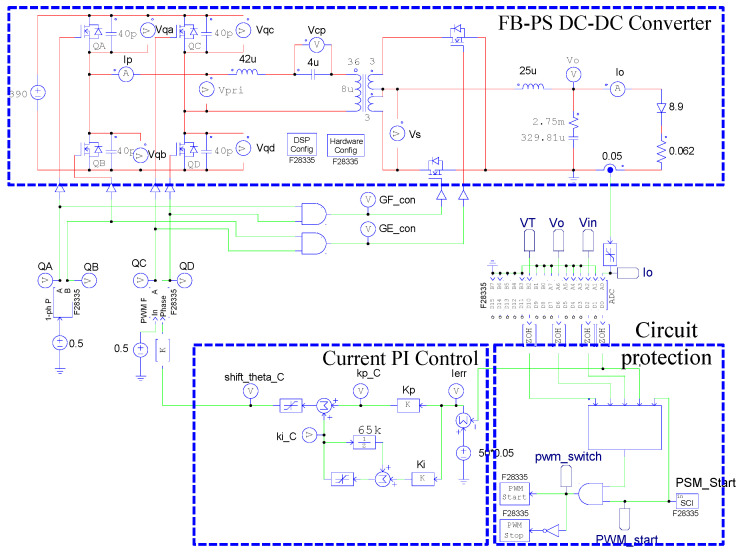
The PSIM simulation model of the proposed LDD on the FB-PS DC-DC converter.

**Figure 16:**
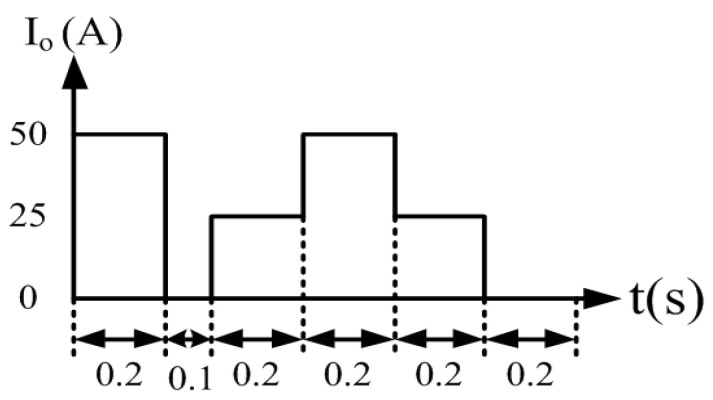
The planned output condition sequence of the LDD.

**Figure 17:**
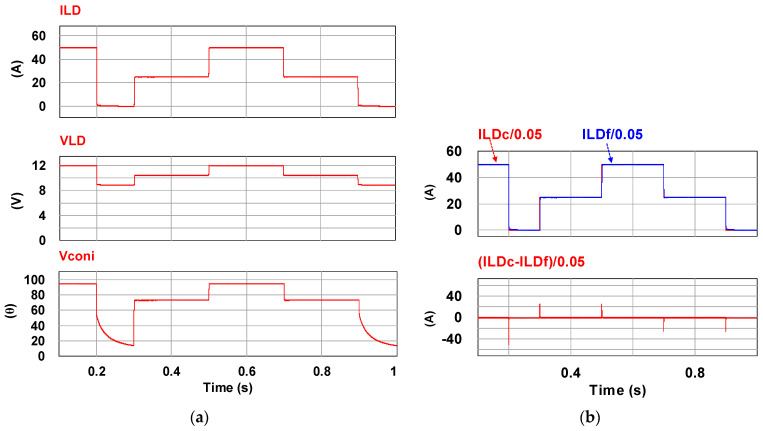
(**a**) Current output/voltage output/phase shift and (**b**) current command and feedback/control error.

**Figure 18:**
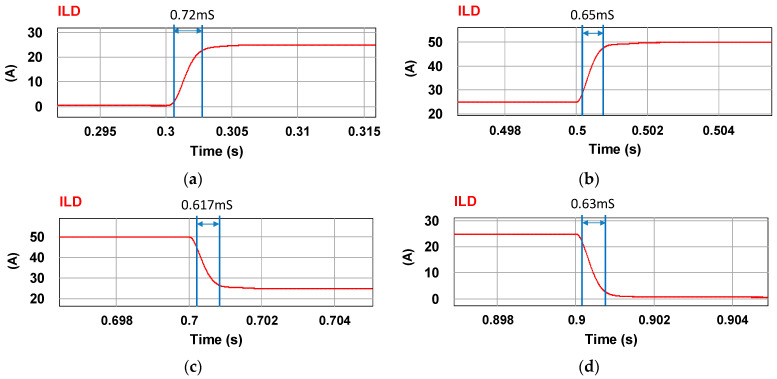
(**a**) 0–25 A rise time, (**b**) 25–50 A rise time, (**c**) 50–25 A fall time, and (**d**) 25–0 A fall time.

**Figure 19:**
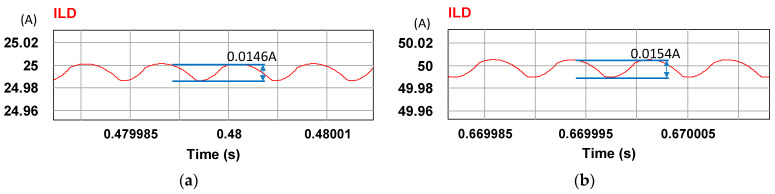
Ripples at (**a**) 25 A and (**b**) 50 A.

**Figure 20:**
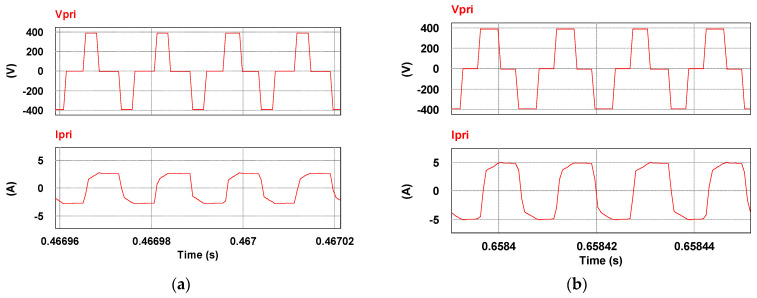
Primary side currents and voltages at (**a**) 25 A and (**b**) 50 A.

**Figure 21:**
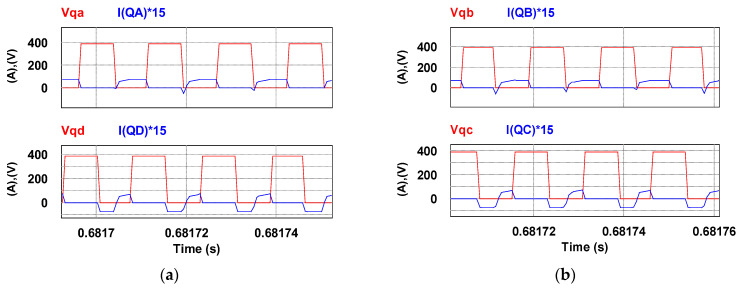
ZVS of the four switches at 50 A output. (**a**) the first pair, and (**b**) the secondary pair.

To verify the correctness of the controller designed for the LDD, a simulation study is used. Figure 15 shows the PSIM simulation model of the proposed LDD on the FB-PS DC-DC converter. The simulated output condition sequence of the LDD is planned as follows: full load (50 A), no load (0 A), half load (25 A), full load, half load, and no load. The time duration for each output condition is 0.2 s. The sequence diagram is shown in Figure 16. Figure 17, Figure 18, Figure 19, Figure 20 and Figure 21 show the simulation results.

## 5. FB-PS DC-DC Converter Implementation

### 5.1. System Configuration and Hardware Test Environment

**Figure 22:**
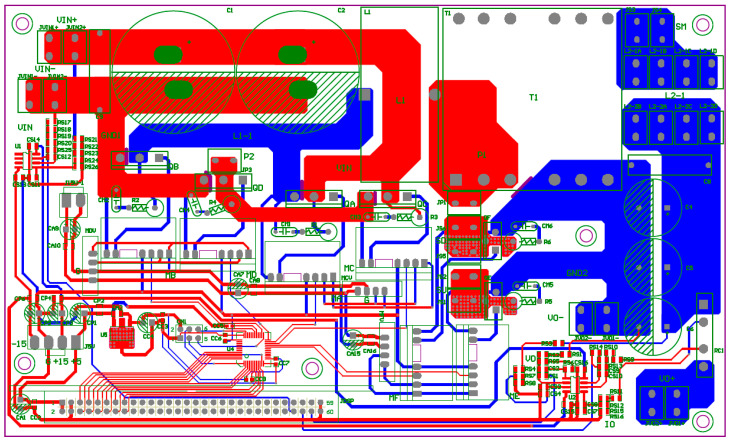
PCB layout of the proposed LDD with an FB-PS DC-DC converter.

**Figure 23:**
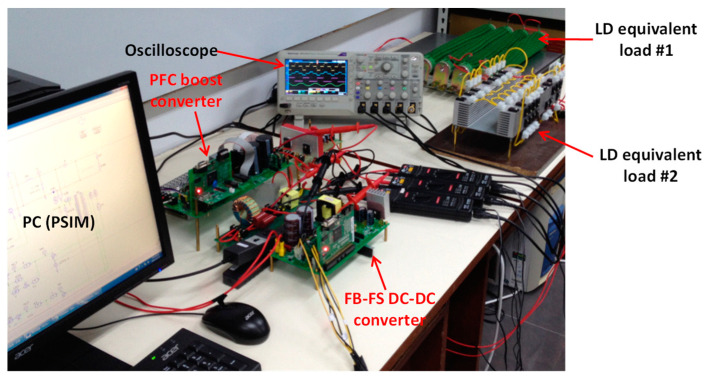
Photograph of the complete LDD experimental system.

To further verify the performance of the proposed LDD, software–hardware integrated implementation and analysis are conducted with a 600 W prototype circuit using a digital signal processor (DSP) as the control core. The arrangement of the hardware system implementation is as follows: a programmable AC power supply is used to emulate a single-phase AC power source from the grid; the digital control unit consists of a PC and a TI TMS320F28335 DSP controller; a multi-output voltage regulator provides ±15 V, 5 V, and 3.3 V power supplies for the ICs and the sensing circuits; a voltage-clamping diode circuit is used to ensure input of 0–3 V for the AD module in the DSP; parameter monitoring is realized with an isolated RS232 communication interface; and the oscilloscope in PSIM is used for real-time observation. It is noted that a commercial high-power LD module is very expensive and dangerous. For security reasons, an equivalent load using a high-current, high-speed diode array (LD equivalent load) whose specifications are the same as the 200 W, 976 nm LD module from DILAS is utilized. A digital oscilloscope is used to measure voltage and current waveforms in real time. Figure 22 presents the full experimental system of the proposed LDD on a FB-PS DC-DC converter. Figure 22 shows the PCB layout, and Figure 23 is a photograph of the experimental hardware.

### 5.2. Experimental Test on LDD Switching Characteristics

**Figure 24:**
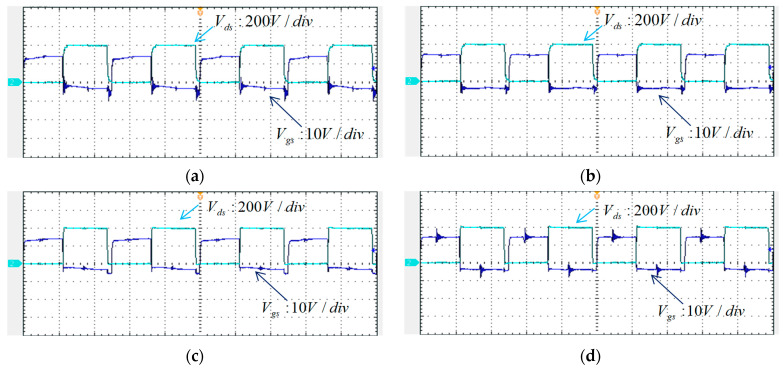
Waveforms of *V_gs_* and *V_ds_* during full-load operation: (**a**) *Q_A_*, (**b**) *Q_B_*, (**c**) *Q_C_*, and (**d**) *Q_D_*.

To perform the full-load open-circuit test, the equivalent LD module using a 0.25 Ω is first carried out. Figure 24 shows the waveforms of the trigger signals of *Q_A_*–*Q_D_* and their respective *V_ds_*. Each time division of the horizontal axis is 5 μs. Channel 1 shows trigger signal, *V_gs_*, and channel 2 shows drain to source voltage, *V_ds_*.

### 5.3. Experimental Test on LDD Driving Performance

**Figure 25:**
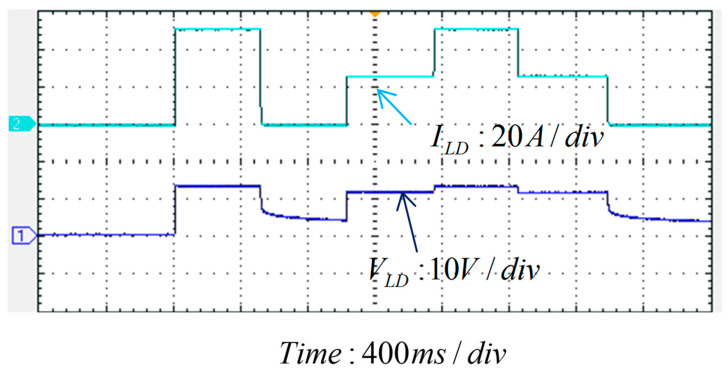
Current and voltage output of the LDD (Time: 400 ms/div).

**Figure 26:**
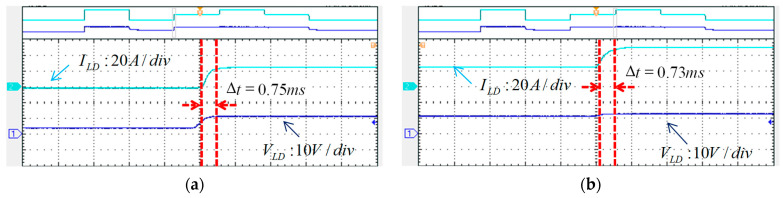

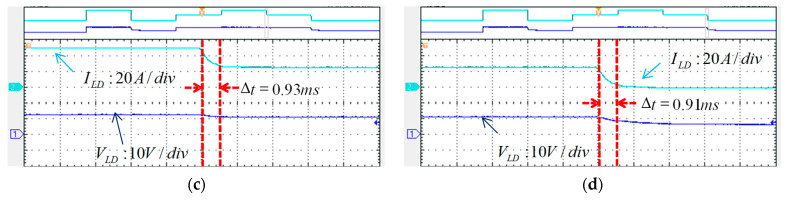
(**a**) 0–25 A current regulation and the rise time, (**b**) 25–50 A current regulation and the rise time, (**c**) 50–25 A current regulation and the fall time, and (**d**) 25–0 A current regulation and the fall time.

For comparison purposes, the LD’s output current commands for the experimental test are the same as those in the simulation scenario. Figure 25 and Figure 26 show the measured waveforms. The results show good similarity to the simulation results, and thus the hardware design and the proposed control scheme are proven feasible and effective.

## 6. Conclusions

With advanced beam integration technology, the power output of fiber laser modules can easily exceed 100 kW, and the development of potential applications of fiber laser and the related driving technologies are highly anticipated. The laser power output of a high-power fiber laser system is usually regulated by an appropriately designed pumping source, whose control quality will directly affect the performance of the fiber laser system. This paper has presented a complete design example of a programmable 600 W continuous wave (CW) LDD that is aimed at driving an LD module with 200 W optical power. The proposed LDD is developed based on a switching mode FB-PS DC-DC converter with a digital control scheme, capable of outputting a 50 A step current command in less than 1 ms. In this study, the design specification of the conversion efficiency set for the proposed LDD is 96% at the rated power. Based on the records obtained from our experimental tests, the conversion efficiency of the proposed LDD system is 96.5% at the rated power (600 W, 50 A/12 V), and a peak efficiency of 97% is observed at the output power of 450 W (about 75% rated power). This paper has explained the detailed working principles of the proposed LDD circuit, the dynamic model derivation of the system, and the quantitative design and verification of the controller. The correctness of the designed controller and overall performance of the LDD are verified through PSIM simulation studies and experimental tests with a hardware prototype circuit to confirm compliance with the design goals. The current control case and related measurements presented are sufficient to demonstrate that the performance of the proposed circuit and control scheme satisfies various control functions and specifications in LDD applications. It is important to note that the circuit configuration and control scheme for tracking the LD current command proposed in this paper provide an important design reference for engineers in related fields.

## Data Availability

Data are contained within the article.

## Abbreviations

$V_{in}$	DC input voltage
$C_{in}$	Input capacitor
$Q_{A,B,C,D,1,2}$	Switching devices of the converter
$D_{A,B,C,D,1,2}$	Intrinsic diode of the switching devices
$C_{A,B,C,D,1,2}$	Drain-source capacitance of the switching devices
$L_{lk}$	Resonant inductor
$V_{P}$	Primary side voltage
$I_{P}$	Primary side current
$T_{1}$	High-frequency transformer
$I_{Q1,2}$	Currents of switches Q_1,2_
$L_{O}$	Output inductance
$C_{O}$	Output capacitor
$V_{o}$	Output voltage
$R_{LOAD}$	Load resistance
$N_{P}$	Turn number of the primary side
$N_{S}$	Turn number of the secondary side
$V_{S1}$	Secondary side upper part voltage
$V_{S2}$	Secondary side lower part voltage
$S_{1,2}$	Secondary side switches
$V_{D}$	Voltage across the body diode of switches
$L_{D}$	Laser diode
$I_{o}$	Output current
$I_{o}^{*}$	Command of the output current
$\hat{V_{in}}$	Small signal of the input voltage
$V_{in}$	Average value of the input voltage
$\hat{i_{in}}$	Small signal of the input current
Vqa,qb, qc,qd	Drain to source voltage of the four switches
$i_{in}$	Input current
N	Turn ratio of the transformer
$\hat{d}$	Small signal of duty
$D_{eff}$	Effective duty
$\hat{i_{L_{o}}}$	Small signal of the output inductor current
$i_{L_{o}}$	Output inductor current
$\hat{V_{L_{o}}}$	Small signal of the output inductor voltage
$V_{L_{o}}$	Output inductor voltage
$R_{ds}$	Switch on resistance
D	Duty cycle
$\hat{i_{C_{o}}}$	Small signal of the output capacitor current
$i_{C_{o}}$	Output capacitor current
$R_{esr}$	Equivalent series resistance
$\hat{V_{o}}$	Small signal of the output voltage
$R_{d}$	Equivalent resistance of diode
$T_{s}$	Switching period
*G_c_*(s)	Current controller
$f_{c}$	Crossover frequency
$k_{c}$	Sensing factor of the output current
ILD	Average current of the LD
VLD	Average voltage of the LD
Vconi	The controlled phase shift
ILDc	LD current command signal
ILDf	Feedback signal of the LD current
Vpri	Primary side voltage
Ipri	Primary side current
I(QA),I(QB), I(QC),I(QD)	Drain to source current of the four switches

## Appendix A

Definitions of variables in the equations for mathematical model derivations:

(A1)
$${\langle V_{L_{o}}\left(t\right)\rangle }_{T_{s}}=V_{L_{o}}+{\hat{V}}_{L_{o}}\left(t\right).$$

(A2)
$${\langle i_{L_{o}}\left(t\right)\rangle }_{T_{s}}=I_{L_{o}}+{\hat{i}}_{L_{o}}\left(t\right).$$

(A3)
$${\langle V_{in}\left(t\right)\rangle }_{T_{s}}=V_{in}+{\hat{V}}_{in}\left(t\right).$$

(A4)
$${\langle d\left(t\right)\rangle }_{T_{s}}=D+\hat{d}\left(t\right).$$

(A5)
$${\langle V_{o}\left(t\right)\rangle }_{T_{s}}=V_{o}+{\hat{V}}_{o}\left(t\right).$$

(A6)
$${\langle V_{C_{o}}\left(t\right)\rangle }_{T_{s}}=V_{C_{o}}+{\hat{V}}_{C_{o}}\left(t\right).$$

(A7)
$${\langle i_{in}\left(t\right)\rangle }_{T_{s}}=I_{in}+{\hat{i}}_{in}\left(t\right).$$

Key equations in the controller design of the FB converter:

(A8)
$$H_{C}\left(2\pi f_{C}\right)=Gain_{H_{C}}∠H_{C}.$$

(A9)
$$\mathrm{boost}angle=PM-∠G_{C}\left(2\pi f_{C}\right)-90{}^{\circ}.$$

(A10)
$$z=2\pi f_{C}/\tan\left(\mathrm{boost}angle\right).$$

(A11)
$$G_{C(temp)}=\left(s+z\right)/s.$$

(A12)
$$Gain_{G_{C}}=1/\left(Gain_{H_{C}}\cdot Gain_{G_{C(temp)}}\right).$$

(A13)
$$G_{C}=\left[{Gain}_{G_{C}}\left(s+z\right)\right]/s$$
